# Genetic Testing Uptake among Ovarian Cancer Survivors in the Genetic Risk Analysis in Ovarian Cancer (GRACE) Study

**DOI:** 10.3390/cancers16142563

**Published:** 2024-07-17

**Authors:** Larissa L. White, Jennifer K. Sawyer, Jamilyn M. Zepp, Yolanda K. Prado, Ana A. Reyes, Mahesh Maiyani, Elizabeth Shuster, Rachel Zucker, Nora B. Henrikson, Alan F. Rope, Sheila Weinmann, Heather S. Feigelson, Jessica Ezzell Hunter

**Affiliations:** 1 Institute for Health Research, Kaiser Permanente Colorado, 16601 East Centretech Parkway, Aurora, CO 80011, USA; jennifer.k.sawyer@kp.org (J.K.S.); mahesh.maiyani@kp.org (M.M.); rachel.x.zucker@kp.org (R.Z.); heather.s.feigelson@kp.org (H.S.F.); 2 Center for Health Research, Department of Translational and Applied Genomics, Kaiser Permanente Northwest, 3800 North Interstate Avenue, Portland, OR 97227, USA; jamilyn.m.zepp@kpchr.org (J.M.Z.); yolanda.k.prado@kpchr.org (Y.K.P.); ana.a.reyes@kpchr.org (A.A.R.); elizabeth.shuster@kpchr.org (E.S.); alan@genomemedical.com (A.F.R.); sheila.weinmann@kpchr.org (S.W.); jehunter@rti.org (J.E.H.); 3 Kaiser Permanente Washington Health Research Institute, 1730 Minor Avenue, Seattle, WA 98101, USA; nora.b.henrikson@kp.org; 4 Genome Medical, 701 Gateway Boulevard, South San Francisco, CA 94080, USA; 5 Genomics, Ethics, and Translational Research Program, RTI International, 3040 East Cornwallis Road, Research Triangle Park, NC 27709, USA

**Keywords:** ovarian cancer, cancer prevention, gynecologic cancer risk reduction

## Abstract

**Simple Summary:**

The improvement in genetic testing uptake for ovarian cancer faces barriers, such as deficiency in access to genetic testing and counseling, lack of referral, and poor follow-up to completion. Genetic risk information can be utilized to guide risk reduction strategies and management during ovarian cancer treatment. The Genetic Risk Analysis in ovarian CancEr (GRACE) study aimed to evaluate the feasibility of retrospective identification (“Traceback”) of both individuals diagnosed with ovarian cancer and their families who could benefit from genetic risk information. The GRACE study findings can assist health care systems to implement and increase genetic testing for survivors of ovarian cancer and other hereditary cancers.

**Abstract:**

Background: Recommendations state all people with ovarian cancers (OCs) receive genetic counseling, but testing uptake is only between 15 and 31%. Those with a prior diagnosis of OC who have not received genetic testing represent a missed opportunity for life-saving genetic risk information. The Genetic Risk Analysis in ovarian CancEr (GRACE) study aimed to evaluate the feasibility of the retrospective identification (“Traceback”) of individuals diagnosed with OC. Methods: This nonrandomized intervention study within two integrated health care systems identified participants with a history of OC between 1998 and 2020 who did not have genetic testing or testing limited to BRCA1/2. Participants received clinical genomic sequencing via a custom 60 gene panel. This study measured the feasibility of the Traceback methodology in OC survivors. Results: The initial cohort included 929 individuals, of which 57% had no prior genetic testing. Of the 302 eligible for recruitment, 88 consented to participate. We were able to outreach 97% of the eligible population using contact information from medical records. The stage at diagnosis was the only factor associated with consent. Of the 78 who returned their saliva sample, 21% had pathogenic/likely pathogenic variants, and 79% had negative results. Conclusion: The GRACE study resulted in a 29% uptake of genetic testing in OC survivors. The time since diagnosis did not have an impact on consent or ability to contact. GRACE can inform the implementation of future Traceback programs, providing guidance on how to prevent and mitigate the burden of OC and other hereditary cancers.

## 1. Introduction

Approximately 23% of all ovarian cancers have a hereditary component, with pathogenic variants in *BRCA1/2* being the most common among epithelial ovarian cancers [1,2,3,4]. In 2024, it is estimated that there will be 19,680 new cases of ovarian cancer and an estimated 12,740 people will die of this disease [5]. Only 30.8% of diagnosed patients survive five years or more due to a late-stage diagnosis and a high recurrence rate due to chemoresistance, making ovarian cancer the leading cause of cancer death of the female reproductive system [5,6,7]. Late-stage ovarian cancer diagnosis is largely due to the absence of safe and effective routine screening options and nonspecific symptomology [8].

Since 2016, National Comprehensive Cancer Network (NCCN) recommendations have stated that all ovarian cases should have a referral to genetic counseling, but the range of testing uptake is only between 15 and 31% [9,10,11]. Barriers to uptake include lack of referral and access to genetic testing and counseling and poor follow-up to completion; this suggests factors impacting uptake occur at both the patient and provider level, extending to gaps in health care system infrastructure [9,12,13]. To address the growing body of evidence that genetic risk extends beyond *BRCA1/2*, NCCN recommended, in 2020, multi-gene panel testing for all individuals diagnosed with epithelial non-mucinous ovarian cancer, including fallopian tube and primary peritoneal cancers [14,15].

The benefits of genetic testing for ovarian cancer extend beyond the diagnosis itself. Genetic testing can identify individual and family risk for related cancers, such as breast cancer and Lynch syndrome [13,16]. This risk information can then be utilized in a provider’s clinical strategy for risk reduction and management, such as surgery or chemopreventive therapies [12,16]. Those with a prior diagnosis of ovarian cancer who have not received genetic testing represent a missed opportunity to receive life-saving genetic risk information for themselves and their families. The Genetic Risk Analysis in ovarian CancEr (GRACE) study aimed to evaluate the feasibility of the retrospective identification (“Traceback”) of individuals diagnosed with ovarian cancer and their families who could benefit from genetic risk information [17].

## 2. Materials and Methods

### 2.1. Study Setting

This nonrandomized intervention study took place within two integrated health care systems, Kaiser Permanente Northwest (KPNW) and Kaiser Permanente Colorado (KPCO). KPCO provides comprehensive health care for approximately 516,000 members in Colorado, and KPNW serves over 600,000 members in Oregon and southwest Washington. The membership of KPNW and KPCO reflect the population in each catchment area. Attrition is low among members, with current annual retention rates of 88% at KPNW and KPCO. Both health systems have tumor registries that track a patient’s entire course of care after a cancer diagnosis and have access to archived pathology specimens from biopsies and surgeries dating back at least 10 years. The electronic health record (EHR) system at both sites includes comprehensive information on diagnoses; medical procedures including surgical pathology; laboratory and imaging tests; genetic testing results; pharmaceutical orders; and notes from clinical encounters. KPNW and KPCO’s EHR was established in 1998. Further details are published elsewhere [17]. The project and all materials were reviewed and approved by the Kaiser Permanente Interregional Institutional Review Board (IRB #1702425-29) and informed consent was required for each participant included in the study. The study covered all genetic testing costs; participants did not receive any additional compensation for their participation.

### 2.2. Study Population

We searched tumor registry data at KPNW and KPCO and identified health plan members with a history of ovarian cancer diagnosed between 1998 and 2020 who either did not have genetic testing or had genetic testing limited to BRCA1/2 and could benefit from testing with a comprehensive cancer gene risk panel. KPCO and KPNW used International Classification of Diseases for Oncology (ICD-O) codes to match inclusion criteria for the cohort. The following codes documented in the EHR between 1998 and 2020 were considered eligible: C481 (Malignant neoplasm of specified parts of peritoneum), C482 (Malignant neoplasm of peritoneum, unspecified), C488 (Malignant neoplasm of overlapping sites of retroperitoneum and peritoneum), C569 (Malignant neoplasm of unspecified ovary), and C570 (Malignant neoplasm of fallopian tube). The following subtypes were considered eligible: endometrioid, clear cell, serous, mucinous, carcinoid, sex cord (stromal), malignant Brenner, peritoneal, and fallopian tube tumors, as well as bilateral teratoma (with family history of teratoma). Other inclusion criteria included female sex in the EHR; no evidence of prior genetic testing, or prior genetic testing for *BRCA1/2* only with a negative result or variant of uncertain significance (VUS); age 18 or older; and a KPNW or KPCO member at the time of ovarian cancer diagnosis. Exclusion criteria included prior diagnosis of a hereditary cancer syndrome, unable to consent in English (KPCO only), unable to provide informed consent, or opted out of research activities. Additionally, the following subtypes were excluded: germ cell, neuroendocrine, benign Brenner, and Krukenberg tumors, as well as sarcomas, uterine cancer in ovary, and lymphoma/leukemia. Borderline ovarian cancer and ovarian cancer of Low Malignant Potential (LMP), including LMP with microinvasion, were also excluded.

### 2.3. Chart Review

Trained research staff conducted a manual chart review to verify the inclusion criteria. Confirmation of ovary, fallopian, and peritoneal cancer was accomplished by identifying the corresponding surgical pathology report based on the tumor registry data. The surgical pathology report was reviewed by the study’s genetic counselor, along with consultation with the study’s physician, to ensure the tumor subtype was eligible for inclusion. The research staff looked for any prior diagnosis of a hereditary cancer syndrome and any prior genetic testing results, which are typically entered into the EHR as scanned documents and queried records where prior genetic testing results may have been captured. Chart review details, contact information, and eligibility status were tracked in REDCap, version 14.4.1 [18,19].

### 2.4. Recruitment Procedure

Starting in March 2021, potential eligible participants were sent a recruitment letter and fact sheet about the study offering genetic testing and counseling for themselves and their families, by both mail and email (if available), which included a personalized link to an electronic consent in REDCap, version 14.4.1 [20]; see Figure S1. Potential participants were also given the option to receive a paper copy of the consent form by mail depending on their preference. Trained research staff followed up by phone with potential participants after two weeks for those who did not respond to the recruitment outreach via mail or e-mail. We defined “active decliners” as people who declined participation during any of the follow-up calls and defined people as “passive decliners” after three failed phone call attempts to reach them.

### 2.5. Genetic Testing

After obtaining informed consent, saliva self-collection kits were mailed from the commercial genetic testing laboratory directly to participants. Participants collected their sample at home and mailed the kit back to the commercial laboratory. As a reminder, study staff called participants who did not return saliva kits after two weeks. The participant was marked as a passive decliner if the saliva kit was not returned after three attempts to reach the participant by phone. A custom 60 gene panel, comparable to the panel patients receive for genetic cancer risk assessment in the clinical setting, was used for clinical genomic sequencing (Table 1). The commercial laboratory performed genetic testing, variant confirmation, and variant interpretation. The results report included both pathogenic and likely pathogenic (P/LP) variants; variants of unknown significance (VUS) were not reported at the request of the study team.

### 2.6. Results Disclosure

Positive test results were returned by phone by the study genetic counselor. The genetic counselor collected family history and relatives’ information during the results disclosure to discuss any changes in care and who would be eligible for cascade testing of the familial variant. The genetic counselor offered to directly contact relatives regarding cascade testing and/or provide a letter the participant could share with their relative to contact the study team. After the call, a letter summarizing the follow-up recommendations and a copy of the test results were mailed or emailed to the participant. Negative results were returned by letter along with a copy of the genetic testing results. All results, both positive and negative, were placed in the participant’s medical record if they were a current or former member of the health plan. The study’s genetic counselor coordinated the next steps in the participant’s care as appropriate, and followed up with family members who expressed interest in testing for the familial variant. Future publications from this study will report on cascade testing among relatives.

### 2.7. Data Analysis

We descriptively measured feasibility using the following metrics: (1) accuracy of tumor registries and pathology reports to identify potential participants with a correct diagnosis, (2) number of eligible participants with available contact information in the EHR, (3) whether time since diagnosis had an impact on available contact information and consent rate, and (4) factors associated with genetic testing uptake (consent). We measured factors associated with uptake using Fisher’s exact tests to account for small cell sizes, and a *p*-value of 0.05 or less was considered statistically significant. All analyses were conducted using SAS, version 9.4 (Cary, NC, USA).

## 3. Results

**Figure 1:**
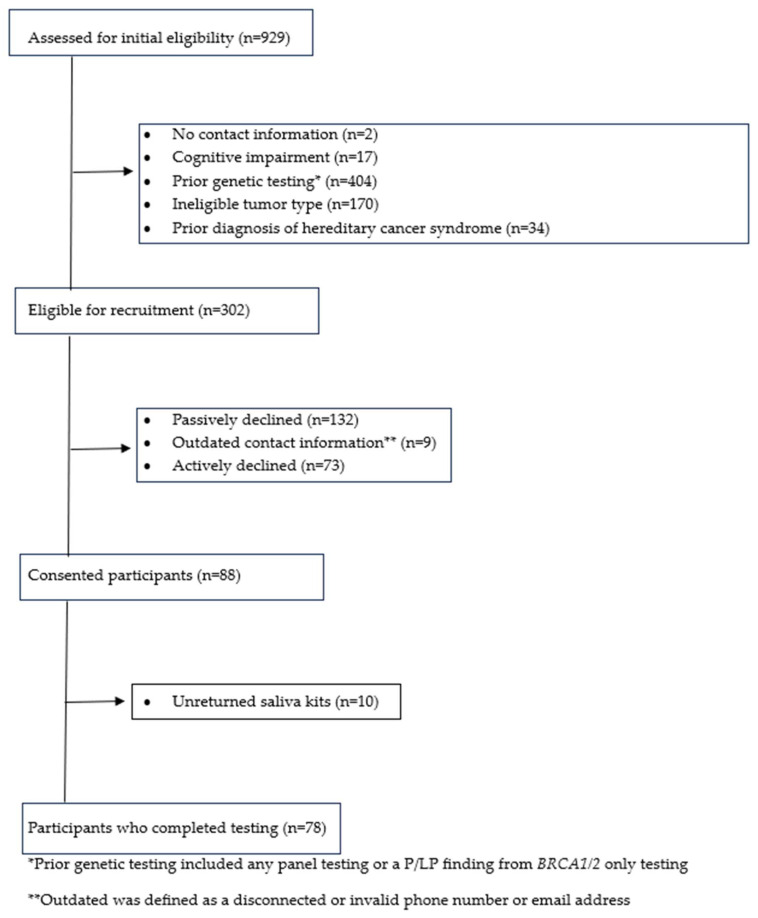
STrengthening the Reporting of OBservational studies in Epidemiology (STROBE) diagram.

Initial eligibility assessment included 929 individuals, of which 43% (*n* = 404) were ineligible due to prior genetic testing, 18% (*n* = 170) had an ineligible tumor type, and 6% had missing contact information (*n* = 2), were cognitively impaired (*n* = 17), or had a prior diagnosis of hereditary cancer syndrome (*n* = 34; Figure 1). Of the 929 initially assessed, 45% had prior panel testing, 11% had *BRCA1/2* testing, 1% had other genetic testing, and 43% had no prior genetic testing. Of the 929 initially assessed, 33% (*n* = 302) were eligible for participation in the study (Figure 1).

Of the 302 eligible for recruitment, 29% (*n* = 88) consented to participate in the study, 44% (*n* = 132) passively declined, 24% (*n* = 73) actively declined, 3% (*n* = 9) had outdated contact information, and 3% (*n* = 10) of consenting participants did not return their saliva kits (Figure 1). Overall, we were able to outreach 97% (*n* = 293) of the eligible population using contact information from the EHR. Reasons for active decline were not systematically captured, but eligible recruits reported to research staff that they had no interest/non-specific reason, no family/biological children, they were managing a serious medical condition or recurrence of cancer either personally or as a caregiver at the time of recruitment, and had privacy concerns as it relates to genetic information.

Of the 302 eligible for recruitment, 61% were diagnosed between the ages of 45 and 64, 52% were diagnosed at stage 1, 56% were 65 years or older at the time of recruitment, 79% were White, and 87% were non-Hispanic/Latino. The eligible and consenting populations were proportionally similar to the overall population of survivors identified in the tumor registry (Table 2). Of those eligible, stage at diagnosis was the only statistically significant factor associated with consent (*p* ≤ 0.0001; Table 2).

**Figure 2:**
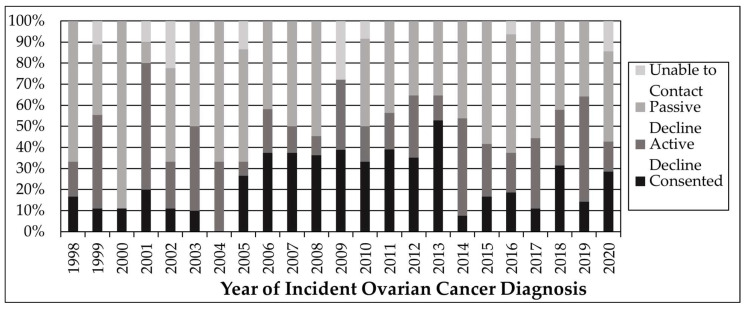
Time since diagnosis, ability to contact, and consent status.

The most successful contact method for recruitment was a combination of letter and email, with a 33% consent rate (Table 3). About 25% of the eligible population preferred to consent electronically. While the prevalence of consent, passive decline, and active decline varied somewhat between eligible participants diagnosed from 1998 to 2020, time since diagnosis was not associated with consent (Figure 2). Time since diagnosis was also not associated with ability to contact, with contact information being available for most eligible participants between 1998 and 2020 (Figure 2).

Of the 78 who returned their saliva sample to perform genetic testing, 21% (*n* = 16) had a P/LP variant result and 79% (*n* = 62) had a negative result. *BRCA1/2* were the most common variants identified from the comprehensive panel, resulting in a positive rate of 13% for genes historically associated with ovarian cancer only (Table 4).

## 4. Discussion

The GRACE study aimed to recruit ovarian cancer survivors who had not yet received genetic testing, representing 57% of our initial study cohort. GRACE study recruitment resulted in a 29% uptake of genetic testing in individuals who initially did not know their genetic risk status, thereby providing potentially life-saving information to their relatives. Recruitment was via email and letter, and saliva kits were mailed directly to participants; thus, no in-person visits were required for participation. Among those who consented, 89% returned their saliva samples, representing a successful genetic testing methodology for ovarian cancer survivors. Contact information was available for 97% of the eligible study population, which included participants who were no longer insured by the health system, and time since diagnosis did not have an impact on consent or ability to contact, further strengthening support for Traceback methods in ovarian cancer survivors. Our findings from this feasibility study translate to several recommendations for future Traceback program implementation.

Our largest proportion of dropout was due to passive decline, showcasing a potential barrier of the Traceback approach. Consent rate was highest for participants who received both electronic and mailed study information, but depending on availability of information in the EHR, some participants received letter or email only. Notes collected on reasons for active decline suggest potential participants had no living family or biological children or had no interest in knowing their genetic risk status, in which case, changes to recruitment methodology are unlikely to have an impact on consent rate. However, it is also possible that participants who passively declined were not expecting to receive an offer for genetic testing, nor were they expecting to consider their past cancer diagnosis, especially those who were no longer insured via KPNW/KPCO. A qualitative study [21] interviewed 70 participants on Traceback genetic testing messaging and uptake. Participants preferred directed, personalized communication and the use of multiple, repetitive modes of outreach, primarily from their physician over other clinicians [21]. Furthermore, the study team [21] hypothesized that direct forms of recruitment may lessen the degree of surprise that participants experience with the Traceback approach. Given these findings and the frequency of passive decline in GRACE, retention for future Traceback studies may be improved by embedding recruitment outreach and reminders via the health system’s respective clinical care unit to improve study corroboration and visibility.

Time since diagnosis did not impact consent rate or ability to contact, which highlights the utility of Traceback approaches to address the health care gap in genetic testing uptake for ovarian cancer survivors, especially those who were diagnosed prior to NCCN guideline implementation. However, the time and staff necessary for manual chart review to identify survivors who are eligible for genetic testing may not be feasible for many health systems. To facilitate genetic testing uptake and to close the gap for genetic testing referral and completion, health systems should consider the implementation of an auto-referral and reminder system to improve genetic testing uptake at or near the time of diagnosis [22,23]. An additional suggestion is for clinicians to reach out to individuals soon after active treatment is complete to inquire about testing at that time.

Relatedly, stage at diagnosis was associated with genetic testing uptake in those who were eligible. While this study cannot address why participants diagnosed at an early stage decided to consent more than those diagnosed at a late stage, about 64% of eligible participants were diagnosed at stages one and two compared to 23% at stages three and four. Given that this is a survivor’s cohort, it is likely that more late stage participants were initially excluded due to vital status. Furthermore, participants diagnosed at an early stage met eligibility criteria more often than those diagnosed at a late stage, suggesting that late-stage participants were excluded due to prior genetic testing or ineligible tumor type. There were no significant differences in other participant-specific factors between those who were eligible and those who consented, indicating that the Traceback approach is unlikely to exacerbate existing health disparities, such as inequities by race/ethnicity.

Our custom gene panel resulted in newly identified P/LP findings for 21% of the tested population, with 13% of P/LP findings specific to ovarian cancer (including *BRCA1/2* and *ATM*). Given that approximately 15% of ovarian cancers are heritable through *BRCA1/2* mutation, our results are slightly lower compared to the current literature on this topic [24,25]. An additional Traceback study [26] saw similar results to the current literature, with 29 consented participants, or 14% of their sample, receiving P/LP findings. However, this study [26] utilized a panel consisting of *BRCA1/2*, *PALB2*, *CHEK2*, and *ATM* only, whereas the GRACE panel was more extensive and included genes not traditionally associated with ovarian cancer, which explains our higher prevalence of P/LP variants overall. Furthermore, the original NCCN guidelines focused more on *BRCA1/2* than the more recently detected variants like *CHEK2*, and it is possible that genetic testing had already occurred for older cases [14,15]. It is unlikely that the exclusion of other ovarian tumors, such as neuroendocrine and germ cell, significantly impacted the identification of P/LP variants, given their rarity [27]. Depending on the aims of future Traceback programs and the state of the literature, researchers and clinicians may want to consider the inclusion of genes associated with cancers other than ovarian to offer a fuller profile of genetic risk assessment and management, especially individuals diagnosed with hereditary breast and ovarian cancer syndrome and Lynch syndrome [4].

Our custom gene panel included both high- and moderate-risk genes for ovarian cancer, as well as other cancers. For many of these genes, there are limited data on cancer risk with no guidelines on clinical actionability [9,14,15,28]. Therefore, it may be difficult to use a singular P/LP variant to assign risk. In many cases, the information from testing for moderate-risk genes does not change clinical management compared to that based on family history alone [9,14,15,28]. Our study’s approach relied on the skills and knowledge of our genetic counselor to interpret a participant’s risk and next steps, given known barriers to genetic risk assessment in clinical care. We highly recommend that other studies and future Traceback programs include a genetic counselor on their team to assist participants in navigating the clinical process after the return of the results.

This study had several strengths. The sample size was sufficient for a rare outcome such as ovarian cancer, making our results statistically generalizable to similar health system populations. We verified ovarian cancer and genetic testing history using both tumor registry data and manual chart review, improving our case definition and application of inclusion/exclusion criteria. Saliva testing was performed by a Clinical Laboratory Improvement Amendments (CLIA)-certified laboratory via third party testing, which is comparable to the current clinical testing procedure for individuals with ovarian cancer in the health care systems in which this study was performed.

We also note some limitations. We could not confirm whether all passive declines were true declines or outdated contact information. However, we did record if contact information was confirmed as outdated. We did not measure differences between the original sampling frame and those who eventually consented, but given the homogeneity of our eligible population vis-à-vis demographics and cancer characteristics, it is unlikely that there are meaningful differences between those who were ineligible and those who consented. However, we recommend future studies examine any potential disparities that the Traceback approach may cause for uninsured/underinsured individuals, as well as those with lower socioeconomic status. Future publications from this study will report on cascade testing among relatives, as well as efforts to provide genetic risk information to families of patients who are deceased.

## 5. Conclusions

The GRACE study findings illustrate the promise of Traceback approaches to provide life-saving information to individuals and their families at increased genetic risk for ovarian cancer. The GRACE study can inform the broad implementation of future Traceback programs across health care systems, providing guidance on how to prevent and mitigate the burden of ovarian and other hereditary cancers.

## Figures and Tables

**Table 1:** Custom gene panel.

Gene List					
*APC*	*CDH1*	*KIT*	*NBN*	*PTCH1*	*SDHD*
*ATM*	*CDK4*	*MAX*	*NF1*	*PTEN*	*SMAD4*
*AXIN2*	*CDKN2A*	*MEN1*	*NF2*	*RAD51C*	*SMARCA4*
*BAP1*	*CHEK2*	*MET*	*NTHL1*	*RAD51D*	*SMARCB1*
*BARD1*	*DICER1*	*MITF*	*PALB2*	*RB1*	*STK11*
*BMPR1A*	*EPCAM*	*MLH1*	*PDGFRA*	*RET*	*TEME127*
*BRCA1*	*FH*	*MSH2*	*PMS2*	*SDHA*	*TP53*
*BRCA2*	*FLCN*	*MSH3*	*POLD1*	*SDHAF2*	*TSC1*
*BRIP1*	*GREM1*	*MSH6*	*POLE*	*SDHB*	*TSC2*
*CDC73*	*HOXB13*	*MUTYH*	*PRKAR1A*	*SDHC*	*VHL*

**Table 2:** Study population characteristics.

Characteristic	Overall (*n* = 929) *N* (%)	Eligible (*n* = 302) *N* (%)	Consented (*n* = 88) *N* (%)	p *
**Age at diagnosis**				0.43
<18	10 (1%)	1 (<1%)	0 (0%)	
18–44	216 (23%)	60 (20%)	20 (23%)	
45–64	481 (52%)	184 (61%)	48 (54%)	
≥65	222 (24%)	57 (19%)	20 (23%)	
**Age at recruitment**				0.12
<18	0 (0%)	0 (0%)	0 (0%)	
18–44	87 (9%)	19 (6%)	9 (10%)	
45–64	357 (39%)	113 (38%)	28 (32%)	
≥65	485 (52%)	170 (56%)	51 (58%)	
**Race**				0.69
American Indian/Alaskan Native	2 (<1%)	1 (<1%)	0 (0%)	
Native Hawaiian/Other Pacific Islander	1 (<1%)	0 (0%)	0 (0%)	
Asian	34 (4%)	11 (4%)	3 (3%)	
Black/African American	25 (3%)	14 (5%)	4 (5%)	
White	764 (82%)	239 (79%)	75 (85%)	
Multiple Races	32 (3%)	5 (2%)	0 (0%)	
Other Race	20 (2%)	7 (2%)	1 (1%)	
Unknown/Not Reported	51 (6%)	25 (8%)	5 (6%)	
**Hispanic/Latino**				0.58
Yes	89 (10%)	29 (10%)	6 (7%)	
No	821 (88%)	262 (87%)	79 (90%)	
Unknown/Not Reported	19 (2%)	11 (3%)	3 (3%)	
**Stage at diagnosis**				<0.0001
0	2 (<1%)	0 (0%)	0 (0%)	
1	374 (40%)	157 (52%)	59 (67%)	
2	83 (9%)	37 (12%)	5 (6%)	
3	190 (20%)	58 (19%)	13 (15%)	
4	71 (8%)	11 (4%)	0 (0%)	
Unknown/Unstaged	209 (23%)	39 (13%)	11 (12%)	

* *p*-value comparing the characteristics between the eligible and consenting populations.

**Table 3:** Consent status by contact method of eligible population.

Contact Method *	Consented (Paper)	Consented (Electronic)	Did Not Consent	Total
Letter (row %)	3 (10%)	1 (3%)	27 (87%)	31
Email (row %)	0 (0%)	12 (23%)	40 (77%)	52
Phone (row %)	0 (0%)	0 (0%)	1 (100%)	1
Letter + Email (row %)	8 (4%)	64 (29%)	146 (67%)	218
Total (row %)	11 (4%)	77 (25%)	214 (71%)	302

* Contact method was based on availability of contact information in the electronic health record (EHR).

**Table 4:** Total newly identified pathogenic/likely pathogenic (P/LP) variants.

Gene	Variant	Reference Sequence ^c^	Diagnosis Year	Diagnosis Age
*ATM*	p.M1? (c.3G>A)	NM_000051.4	2006	65
*APC* ^a,b^	p.I1307K (c.3920T>A)	NM_000038.6	2018	34
*BARD1* ^b^	c.2300_2301delTG	NM_000465.2	2013	61
*BRCA1*	c.3756_3759delGTCT	NM_007294.4	2013	54
*BRCA1*	p.V1736A (c.5207T>C)	NM_007294.4	2006	48
*BRCA2*	c.5073dupA	NM_000059.4	2010	57
*BRCA2*	c.3847_3848delGT	NM_000059.4	2011	60
*CHEK2*	p.H143Y (c.427C>T)	NM_007194.4	2007	55
*DICER1*	p.R688* (c.2062C>T)	NM_177438.3	2013	23
*HOXB13* ^a,b^	p.G84E (c.251G>A)	NM_006361.6	2007	47
*MITF* ^a,b^	p.E318K (c.952G>A)	NM_000248.3	2007	51
*MUTYH* ^a,b^	p.G396D (c.1187G>A)	NM_001128425.1	2006	49
*MUTYH* ^a,b^	p.G396D (c.1187G>A)	NM_001128425.1	2013	68
*PMS2*	c.1040delA	NM_000535.7	2013	52
*RAD51D*	c.896_*505del761insT	NM_002878.4	2006	83
*SDHB* ^a,b^	p.L111V (c.331C>G)	NM_003000.3	2009	62

^a^ Genetic test result not related to personal or family cancer history; ^b^ gene not historically associated with ovarian cancer risk; ^c^ matched annotation from the National Center for Biotechnology Information (NCBI) and European Molecular Biology Laboratory-European Bioinformatics Institute (EMBL-EBI) (MANE) reference sequence, version 1.3.

## Data Availability

The data underlying this article cannot be shared publicly due to the privacy of individuals and health system members that participated in the study. The derived data will be shared on reasonable request to the corresponding author.

## Supplementary Materials

The following supporting information can be downloaded at: https://www.mdpi.com/article/10.3390/cancers16142563/s1, Figure S1: Fact sheet.
