# Supplementary material for: Genetic Testing Uptake among Ovarian Cancer Survivors in the Genetic Risk Analysis in Ovarian Cancer (GRACE) Study
Source: Cancers (Basel). 2024 Jul 17;16(14):2563. doi: 10.3390/cancers16142563 (PMC11274893; doi:10.3390/cancers16142563)
Supplement: Supplementary file 1 [file cancers-16-02563-s001.zip › cancers-3040340-supplementary.pdf]

KAISER PERMANENTE®

Genetic Risk Analysis in Ovarian Cancer (GRACE) Study

1 in 190

People have a gene change that increases the risk of ovarian cancer

What is inherited cancer?

While most cancers happen by chance, some cancers run in families. These inherited cancers happen because a person is born with a gene change that raises their risk for cancer. About 15% of ovarian cancers are inherited.

What should I know about inherited cancer?

People with a gene change that increases their risk of cancer have a higher risk of developing cancer at a younger age. They may want to talk to their doctor about how to prevent cancer or start screening for cancer earlier than usual.

How are family members affected?

If a person has a gene change that increases their risk of cancer, their blood relatives could have a genetic test to find out if they have also have a higher risk.

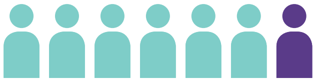

1 in 7

cases of ovarian cancer are because of a gene change

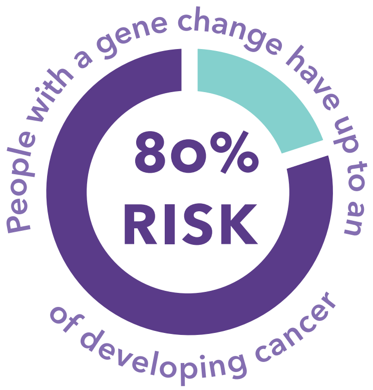

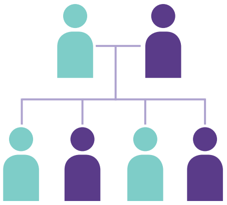

If a person has an inherited gene change, their parents, children, and siblings have a 50% chance of having this gene change too

What is the GRACE study?

GRACE is a study that aims to find people who carry a gene change that increases their risk for cancer. We will provide genetic testing to people who had ovarian cancer. If the person carries a gene change, we will provide genetic testing to their relatives.

For more information about the GRACE study, please contact the study team at chr_grace@kpchr.org or 503.335.2400.

GRACE
Genetic Risk Analysis in Ovarian Cancer

CHR 60750 10/06/2020
